# Effect of chewing on dental patients with total denture: an experimental study

**DOI:** 10.1186/2193-1801-2-40

**Published:** 2013-02-08

**Authors:** Mahmut Tokmakci, Mustafa Zortuk, Musa Hakan Asyali, Yildiray Sisman, Halil ibrahim Kilinc, Elif Tarim Ertas

**Affiliations:** 1 Department of Biomedical Engineering, Faculty of Engineering, Erciyes University, 38039 Kayseri, Türkiye; 2 Department of Prosthodontics, Faculty of Dentistry, Erciyes University, 38039 Kayseri, Türkiye; 3 Department of Electrical and Electronics Engineering, Faculty of Engineering, Antalya International University, 07190 Antalya, Türkiye; 4 Department of Oral Diagnosis and Radiology, Faculty of Dentistry, Erciyes University, 38039 Kayseri, Türkiye

**Keywords:** Edentulous patients, Total denture, Electromyography, Wavelet analysis

## Abstract

In this study, we have explored the prospect of assessing and following level of total denture adaptation by use of EMG signals recorded during gum chewing. Total of 14 edentulous patients, 6 women and 8 men, with an average age of 63±9 years, were recruited. Separate EMG recordings were obtained from left and right temporalis and masseter muscles of the patients for a period of 10 seconds, while they were chewing a sugar-free gum on their left and right sides. EMG recordings were repeated at three times: before, right after, and six months after the placement of the denture. We have tried to standardize environmental and individual factors during EMG recordings. The EMG data have been pre-processed and analyzed using Discrete Wavelet Transform (DWT) and obtained features were statistically evaluated using the paired sample t-test. Chewing activity on the right and left side is analyzed by making comparisons of muscle activity between before and right-after cases and before and six-months-after denture fixation cases. A comparison between right and left side mastication is also made at different time points. We have suggested and implemented a new test and comparison procedure in order to assess adaptation to denture fixation using EMG analysis. In this study, the results indicate that DWT based EMG analysis is instrumental in evaluating denture adaptation and as time progresses the adaptation to denture and hence chewing efficiency increases in patients with total denture replacement.

## Introduction

Study of mechanical and electrical features of muscles have become focal point for researchers in dentistry as well as many medical disciplines including physiology, biomechanics, neurologic sciences, physical medicine and rehabilitation (Okeson 1998; Hatch et al. 2001; Wayler & Chauncey 1983). One of the objectives of the prosthesis rehabilitation is to provide the masticator function as best as possible. This study aims at determining and following the level of total denture adaptation by use of Electromyography (EMG) signals.

EMG analyses are used in order to evaluate how fixed or removable dentures affect the performance of chewing muscles (Chauncey et al. 1984; Julien et al. 1996a; Tumrasvin et al. 2006). Research has shown that EMG is an important tool in determining how prostheses contribute to stomatologic system in terms of physiology and functionality (Julien et al. 1996b; Akeel et al. 1992; Kemsley et al. 2003; Hagberg 1986). There also exist several studies in the literature that aim at assessing the masticatory muscle function using color-changing chewing gums (Liedberg & Owall 1995; Hayakawa et al. 1998; Prinz 1999). Studies by (Kemsley et al. 2003) and (Hagberg 1986) have shown that masseter and temporalis anterior muscles are preferred in EMG studies of chewing function. In this study, we combine these two ideas, namely evaluation of EMG signals acquired during gum chewing in assessing the level of total denture adaptation.

(Winnberg & Pancherz 1983), (Kibana et al. 2002) reported the head posture affects all the parameters of EMG while chewing. The same authors also conclude that, with standardization of environmental factors such as light, heat, noise, and the time period in a day more homogeneous results could be obtained. Therefore, to determine the muscle activity during chewing function more objectively, we have tried to give much attention to standardize the environmental and individual factors such as having the records at the same time (13:30–16:00), in the same sitting position and with a stable head posture.

Artificial food as well as natural food is used while evaluating chewing function. Plesh et al. preferred chewing gum as the test material because of the uniform density during chewing cycle (Plesh et al. 1986). At the first thought, natural test foods may be considered advantageous because of their consumption in daily lives and familiarity with them. However, this issue can vary according to seasonal and geographical factors. In order to avoid this kind of variability in assessing chewing function, some researchers report that the use of synthetic food is a good alternative (Albert et al. 2003; Fontijn-Tekamp et al. 2000; Mizumori et al. 2003; Stagter et al. 1993). Further, as some of the natural food may be consumed during the EMG recording, the chewing activity may show relatively large fluctuations. On the contrary, in case of chewing gums, due to the fact the test food is not consumed, we are more likely to obtain more accurate measurements of chewing activity.

(Hayasaki et al. 2003) and (Shiga et al. 2001) also used chewing gum in their studies. Because masticator efficiency is a good measure of chewing function, a material with uniform properties that can be reliably reproduced would provide an ideal test bolus for the scientific study of masticator effectiveness (Compagnon et al. 1999). In some previous studies, methods using gum have been developed to simplify and standardize the test procedures (Blissett et al. 2007; Mazari et al. 2007; Prinz & Heath 2000). In this study, we offered sugar-free chewing gum to participants, as it can be applied easily and being sugar- and/or sweetener-free it will not cause salivary stimulation that can influence the mastication function (Anastassiadou & Heath 2001). Further, with its uniform properties in terms of weight and shape, this type of test food has also helped us standardize EMG measurements.

In order to evaluate mastication activity of total denture patients, four channels of EMG signals are recorded from right and left masseter and temporalis muscles at three different times: “before”, “right-after”, and “six-months-after” denture fixation. The EMG signals have been processed by using wavelet analysis to extract several level wavelet parameters. These parameters or coefficients were then compared statistically with the “paired sample t-test”.

## Materials and methods

### Subjects

In order to make denture adaptation comparisons using EMG signals in a simple manner, we selected edentulous patients who naturally chew on their right side. We recruited total of 14 such subjects (6 women and 8 men) who were admitted to the Faculty of Dentistry at Erciyes University from Cappadoccia region of Turkey between years of 2007 and 2008. Informed consent was obtained from subjects prior to the study. The average age of our edentulous patients was 63 ± 9 years. Oral examination was carried out by two dentists, according to the WHO method (World Health Organization 1997) using lighting and a mouth mirror.

Before fabrication of the denture, simultaneous EMG recordings were obtained from both anterior temporalis and masseter muscles bilaterally (total of 4 locations or muscles) of subjects while they were chewing on the left and right sides separately. Right after finishing the denture with routine techniques, and six months after the denture usage, activation of the muscles during chewing on right and left sides and were evaluated again using EMG.

During EMG data collection, which lasted for 10 seconds, we asked the participants to chew Falım^™^ (Intergum Co., Istanbul, Turkiye) brand, 1.3 g rectangular shaped portions of sugar free chewing gums.

### Electromyography recordings

Electrical activity of right and left masseter and temporalis muscles were evaluated for the study. During recording process, the patients were seated on a chair with their heads in upright position. While placing the electrodes, the patients were asked to hold their jaws tightly closed so that we could palpate the muscles for a proper placement. Electrode sites were cleaned with isopropyl alcohol soaked pads, before placing the electrodes. In order to reduce the contact impedance between the surface electrodes and the skin, we used conductive electrode-gel. All of the electrodes were attached to the skin with medical plasters.

**Figure 1:**
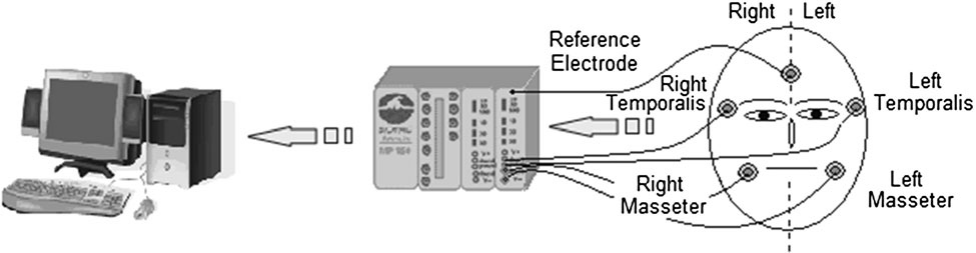
**Four channels measurement arrangement for EMG recording process (two electrodes for temporalis muscles, two electrodes for masseter muscles, and one reference electrode on the forehead).**

Bipolar surface electrodes (Biopac Systems, Inc. Santa Barbara, California) filled with a conductive gel were placed on both the right and left masseter and anterior temporalis muscles as shown in Figure 1. The subjects were asked to refrain from moving their head or talking during the EMG recording.

The EMG signals are band-pass filtered (10–500 Hz), amplified with a gain of 2000 (amplifier model EMG100B, Biopac Systems Inc., Santa Barbara, California) and converted to digital signals at a sampling rate of 2000 samples per second, using a MP100A-CE (Biopac Systems Inc., Santa Barbara, California). Surface EMG recordings are obtained from the left and right anterior temporalis muscle, the left and right masseter muscle and from the left and right digastric muscle using 4 channel PC based EMG apparatus, for simultaneous recording of the myoelectrical activity. Ag/AgCl disc electrodes with 1 cm diameter were placed 2 cm apart in the main direction of the muscle fibers.

The collected raw EMG signals were pre-processed using rectification and low-pass filtering (De Luca 1997). The rectification is simply done by just taking or retaining the positive half of the signal. The envelope detection or smoothing is done by low-pass filtering. After these pre-processing steps, the resultant EMG signal resembles the force signal generated by the underlying muscles as a function of time. For the muscles involved in this study, a low-pass filter of about 5 Hz has been found to produce an envelope similar in shape to the force signals generated by the muscle. We have then employed discrete wavelet transform, which is described next, to extract information (i.e., to compute some features) from these processed EMG signals.

### Wavelet analysis of EMG recordings

**Figure 2:**
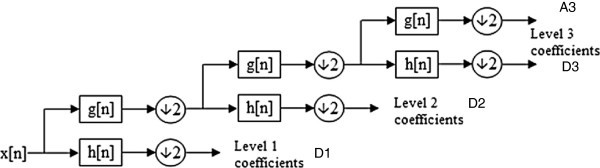
**Sub-band decomposition of discrete wavelet transform (DWT) implementation; x[n] is input signal, g[n] is the high-pass filter, h[n] is the low-pass filter, and C is coefficient matrix which is decomposition of wavelet coefficients with three level ‘Db3’ (The abbreviations A and D respectively stand for Approximation and Detail).**

In this study, EMGs were decomposed or analyzed with ‘Db3’ Daubechies Wavelets to the third level as shown in Figure 2. Third detail signals and third approximation signal at the third level were reconstructed from the coefficient matrices. All calculations were done using Matlab Software Release 2007b (The MathWorks, Inc., Natick, MA).

The Wavelet Transform (WT) is designed for time-frequency analysis of non-stationary signals. It involves representing signals in terms of simple, fixed building blocks termed wavelets. These building blocks are actually a group of functions, which are derived from a single generating function called the mother wavelet by translation and scaling operations in time (Daubechies 1990). Scaling summarizes the mother wavelet and translation shifts it along the time axis and so it has a varying window size, being broad at low frequencies and narrow at high frequencies, thus leading to an optimal time-frequency resolution in all frequency ranges. The WT can be classified into continuous and discrete. The Discrete Wavelet Transform (DWT) is often used because of a considerable effort and a vast amount of data being necessary to calculate a continuous wavelet of a signal.

All wavelet transforms can be specified in terms of a low-pass filter h as shown in Figure 2, which satisfies the standard quadrature mirror filter condition:1

where *H*(*z*) denotes the *z*-transform of the filter *h*. Its complementary high-pass filter can be defined as,2

A sequence of filters with increasing length (indexed by *i*) can be obtained.34

with the initial condition *H*_0_(*z*) = 1. It is expressed as a two-scale relation in time domain5

where the subscript [ ]_*↑*m_ indicates the up-sampling by a factor of *m* and *k* is the equally sampled discrete time.

The normalized wavelet and scale basis functions *φ*_*i*,*l*_(*k*),  *ψ*_*i*,*l*_(*k*) can be defined as,6

where the factor 2^i/2^ is an inner product normalization, *i* and *l* are the scale and translation parameter, respectively. The discrete wavelet transform decomposition can be described as7

where *A*_(*i*)_(*l*) and *D*_*i*_(*l*) are the approximation and detail coefficients at resolution *i*, respectively (Girault et al. 2000; Zhang et al. 2001). We have chosen to use A1, A2, and A3, namely approximation coefficients, because these reflect the main characteristics of the signal under study, whereas the detail coefficients D1, D2, and D3 essentially represent the noise in the signal at various levels.

## Results

**Figure 3:**
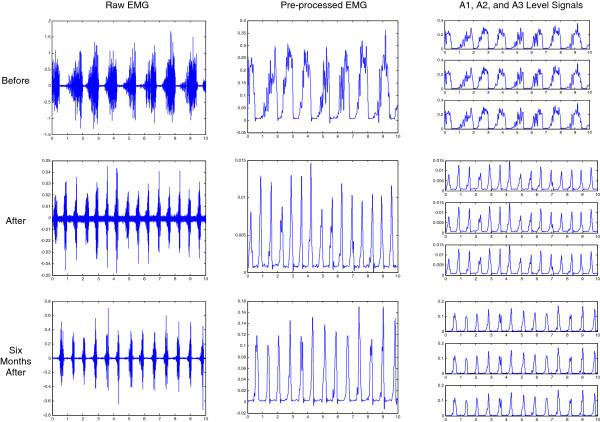
**EMG signals obtained from subject #6, during chewing on the right side.** The rows in the figure correspond to the recording times of “before”, “right-after”, and “six-months-after” denture fixation. Whereas the columns correspond to raw, pre-processed, and A1 (upper plot), A2 (middle plot), and A3 (lower plot) level wavelet components of pre-processed EMG signals.

We display sample EMG signals (obtained from subject #6, during chewing on the right side) and its processing steps, in Figure 3. There are three rows of plots in the figure; upper, middle, and bottom rows correspond to the recording times of “before”, “right-after”, and “six-months-after” denture fixation, respectively. The left panels show the raw EMG signal, whereas the middle panels depict the rectified and low-pass filtered, i.e. pre-processed, EMG signals. The right panels show the decomposition of the pre-processed EMG signals into A1 (upper plot), A2 (middle plot), and A3 (lower plot) level wavelet signals. These component signals are obtained by simply multiplying estimated DWT approximation coefficients by the wavelets at the corresponding levels.

DWT coefficients obtained from pre-processed EMG recordings for different cases were compared statistically using the “paired sampled t-test.” We used the paired sample t-test, rather than the independent sample t-test, as the groups of DWT parameters being compared were obtained from the same set of subjects under different conditions. The p-values less than 0.05 were considered as statistically significant.

In Table 1, we compare results of wavelet based analysis of EMG records from chewing process on the right side for “before” and “right-after” denture fixation cases. We observe that for mastication on the right side, a significant change between left masseter and temporalis muscle activities was observed, however no such significant change was observed between right masseter and temporalis muscle activities. Keeping in mind that our patients are using their right side for mastication, this result underlines the fact that the patients used masticate on the right side when they were edentulous and therefore after the denture is made the same activity is observed in the right side muscles. However, the change in the left side muscles during right side mastication shows that the mastication forces have been distributed due to the obtained occlusion.

**Table 1:** **Comparison of EMG wavelet parameters for chewing process on the right side between “before” and “right after” denture fixation**

		Before			Right-After			Significance (p-values)		
		A1	A2	A3	A1	A2	A3	p-A1	p-A2	p-A3
Right Masseter	Mean	0.0443	0.0438	0.0390	0.0345	0.0342	0.0297	0.4730	0.4744	0.4090
	SD	0.0248	0.0246	0.0219	0.0354	0.0351	0.0282			
Left Masseter	Mean	0.0389	0.0385	0.0340	0.0162	0.0161	0.0147	**0.0026**	**0.0027**	**0.0035**
	SD	0.0209	0.0207	0.0184	0.0051	0.0051	0.0048			
Right Temporalis	Mean	0.0454	0.0447	0.0397	0.0328	0.0327	0.0310	0.3264	0.3499	0.4757
	SD	0.0227	0.0220	0.0174	0.0087	0.0086	0.0074			
Left Temporalis	Mean	0.0450	0.0444	0.0393	0.0222	0.0221	0.0206	**0.0251**	**0.0258**	**0.0327**
	SD	0.0333	0.0327	0.0281	0.0106	0.0106	0.0101			

Similarly, in Table 2, we report comparison results from chewing process on the right side for “before” and “six-months-after” denture fixation cases. We note that, for the right side there is no significant change as expected, whereas for left side mastication there are significant changes among wavelet parameters obtained for “before” and “six months after” denture fixation. This indicates that the adaptation is properly functioning on the left side.

**Table 2:** **Comparison of EMG wavelet parameters for chewing process on the right side between “before” and “six months after” denture fixation**

		Before			Six-Months-After			Significance (p-values)		
		A1	A2	A3	A1	A2	A3	p-A1	p-A2	p-A3
Right Masseter	Mean	0.0443	0.0438	0.0390	0.0691	0.0680	0.0580	0.0854	0.0879	0.1170
	SD	0.0248	0.0246	0.0219	0.0150	0.0149	0.0140			
Left Masseter	Mean	0.0389	0.0385	0.0340	0.0869	0.0861	0.0741	**0.0085**	**0.0087**	**0.0109**
	SD	0.0209	0.0207	0.0184	0.0092	0.0092	0.0083			
Right Temporalis	Mean	0.0454	0.0447	0.0397	0.0543	0.0529	0.0443	0.0740	0.0783	0.1050
	SD	0.0227	0.0220	0.0174	0.0188	0.0186	0.0159			
Left Temporalis	Mean	0.0450	0.0444	0.0393	0.1043	0.1021	0.0853	**0.0301**	**0.0309**	**0.0344**
	SD	0.0333	0.0327	0.0281	0.0097	0.0097	0.0086			

In Tables 3 and 4, chewing activity on the left side is analyzed by making comparisons for “before and right-after” and “before and six-months-after” denture fixation cases respectively. Since the left side was not used before denture fixation by our subjects, we expected more changes in muscle activity on this side, as a sign of adaptation to denture. Confirming this expectation, we note that there are statistically significant changes for all four observed muscle activities for both comparisons.

**Table 3:** **Comparison of EMG wavelet parameters for chewing process on the left side between “before” and “right after” denture fixation**

		Before			Right-After			Significance (p-values)		
		A1	A2	A3	A1	A2	A3	p-A1	p-A2	p-A3
Right Masseter	Mean	0.0397	0.0392	0.0348	0.0216	0.0214	0.0191	**0.0322**	**0.0338**	**0.0311**
	SD	0.0206	0.0204	0.0186	0.0247	0.0246	0.0213			
Left Masseter	Mean	0.0404	0.0400	0.0355	0.0165	0.0164	0.0151	**0.0005**	**0.0006**	**0.0008**
	SD	0.0209	0.0208	0.0189	0.0119	0.0118	0.0109			
Right Temporalis	Mean	0.0414	0.0407	0.0364	0.0300	0.0299	0.0287	**0.0060**	**0.0077**	**0.0198**
	SD	0.0196	0.0190	0.0155	0.0116	0.0114	0.0099			
Left Temporalis	Mean	0.0489	0.0482	0.0423	0.0305	0.0303	0.0282	**0.0390**	**0.0414**	**0.0455**
	SD	0.0314	0.0309	0.0266	0.0215	0.0214	0.0196

**Table 4:** **Comparison of EMG wavelet parameters for chewing process on the left side between “before” and “six months after” denture fixation**

		Before			Six-Months-After			Significance (p-values)		
		A1	A2	A3	A1	A2	A3	p-A1	p-A2	p-A3
Right Masseter	Mean	0.0397	0.0392	0.0348	0.0706	0.0695	0.0593	**0.0101**	**0.0106**	**0.0158**
	SD	0.0206	0.0204	0.0186	0.0090	0.0090	0.0083			
Left Masseter	Mean	0.0404	0.0400	0.0355	0.0658	0.0650	0.0562	**0.0319**	**0.0331**	**0.0452**
	SD	0.0209	0.0208	0.0189	0.0106	0.0105	0.0094			
Right Temporalis	Mean	0.0414	0.0407	0.0364	0.0656	0.0634	0.0521	**0.0052**	**0.0055**	**0.0095**
	SD	0.0196	0.0190	0.0155	0.0057	0.0056	0.0049			
Left Temporalis	Mean	0.0489	0.0482	0.0423	0.0754	0.0737	0.0623	**0.0176**	**0.0184**	**0.0232**
	SD	0.0314	0.0309	0.0266	0.0159	0.0158	0.0137			

In Table 5, comparison between right and left side mastication in terms of significance (p-values) for “before”, “right-after”, and “six-months-after” denture fixation cases are presented. We note that for “before” case there are significant changes on all muscle activities, whereas for “right-after” and “six-months-after” cases there are no significant changes between right and left side mastication. This indicates that before the use the left side for mastication, there is differential activation of all observed muscles during right and left mastication. However, as the patients start and continue to use the dentures, they adapt both physically and psychologically and eventually an equal mastication distribution is obtained on all observed muscles. Despite the use of different analytical techniques, this result is confirmed by the literature (Mishellany-Dutour et al. 2008).

**Table 5:** **Comparison of right and left side mastication in terms of significance (p-values) for “before”, “right after”, and “six months after” denture fixation**

	Before			Right-After			Six-Months-After		
	p-A1	p-A2	p-A3	p-A1	p-A2	p-A3	p-A1	p-A2	p-A3
Right Masseter	**0.0068**	**0.0057**	**0.0069**	0.5256	0.5499	0.4958	0.2790	0.2385	0.2567
Left Masseter	**0.0013**	**0.0012**	**0.0013**	0.7837	0.7765	0.7962	0.9566	0.8980	0.7987
Right Temporalis	**0.0120**	**0.0121**	**0.0118**	0.5574	0.5673	0.5468	0.3858	0.4223	0.3233
Left Temporalis	**0.0038**	**0.0034**	**0.0039**	0.1115	0.1218	0.1323	0.3338	0.3542	0.3145

In Tables 1, 2, 3 and 4, SD denotes Standard Deviation and in Tables 1, 2, 3, 4 and 5, A1, A2, and A3 refer to the amplitude values of wavelet levels.

## Discussion and conclusion

In this study, the effect of complete dentures on the chewing muscle activity was investigated with the analysis of EMG collected during chewing on the right and left side, from masseter and temporalis muscles which are known to be more effective in assisting mastication. In an attempt to make denture adaptation comparisons using EMG signals in a simple manner, we have chosen to work with edentulous patients who naturally chew on their right side.

In order to evaluate mastication activity of total denture patients, four channels of EMG signals are recorded from right and left masseter and temporalis muscles at three different times: “before”, “right-after”, and “six-months-after” denture fixation. The EMG signals have been processed by using wavelet analysis to extract several level wavelet parameters. These parameters or coefficients were then compared statistically with the “paired sampled t-test.”

When we examine the results presented in Tables 1, 2, 3, 4 and 5, we deduce the following points:

1. For “before and right-after” and “before and six-months-after” denture fixation comparison of mastication on the right side, there is a significant difference between left masseter and temporalis muscle activities was observed, whereas there is no such significant difference for the right side muscles. Since the patients used to masticate on the right side when they were edentulous, after the denture is made, same activity is observed on the right side muscles. However, the change in the left side muscles during right side mastication shows that the left side mastication is also in effect due to the obtained occlusion by the denture.
2. For “before and right-after” and “before and six-months-after” denture fixation comparisons of mastication on the left side, there are statistically significant changes for all four observed muscle activities for both comparisons. Since the left side was not used before denture fixation by our subjects, we expected such changes in muscle activity on the left side, as a sign of adaptation to denture.
3. For “before”, “right-after”, and “six-months-after” comparison of between right and left side mastication, there is a significant difference only for the “before” case. This may signify that before the use the left side for mastication, there was differential activation of all four observed muscles during right and left side chewing. However, as the patients start using and adapting to the dentures, a homogenous mastication distribution is obtained on the muscles.
4. For all wavelet coefficients A1, A2, and A3 significant or non-significant decision are consistently in agreement. This observation confirms that the wavelet analysis is instrumental in extracting information from EMG signals. Making comparisons using raw EMG signals is a formidable task. Instead, we have first processed EMG signals to obtain their envelope, which represents the force produced by the underlying muscle in a more accurate manner, and obtained several wavelet parameters from this EMG-envelope.

These observations confirm that wavelet based EMG analysis is instrumental in evaluating denture adaptation for patients with total denture replacement and denture adaptation and hence chewing efficiency increases with time.

In conclusion, we have suggested and implemented a new test and comparison procedure in order to assess adaptation to denture fixation using EMG analysis. Using chewing gum as a test food is an important aspect of this approach.

In this study, for ease of analysis, we have chosen to work with edentulous subjects who naturally chew on their side as our study group. In a future study, we are going to evaluate the efficacy of this approach in different scenarios, where the edentulous subjects to be selected could have different characteristics, in terms of their natural chewing preferences.
